# Antimigraine medication use and associated health care costs in employed patients

**DOI:** 10.1007/s10194-011-0405-6

**Published:** 2011-11-30

**Authors:** Jun Wu, Mary D. Hughes, Matthew F. Hudson, Peggy J. Wagner

**Affiliations:** 1 South Carolina College of Pharmacy, University of South Carolina, Health Sciences Administration Bldg (MIPH), 701 Grove Road, Greenville, SC 29607 USA; 2 Division of Neurology, Department of Medicine, Greenville Hospital System, University Medical Group, 701 Grove Road, Greenville, SC USA; 3 Greenville Hospital System, University Medical Center, 701 Grove Road, Greenville, SC USA; 4 Department of Family and Community Medicine, University of South Carolina, 701 Grove Road, Health Sciences Administration Bldg (MIPH), Greenville, SC USA

**Keywords:** Migraine, Antimigraine medication use, Health care costs, Employed patients

## Abstract

Migraine is under diagnosed and suboptimally treated in the majority of patients, and also associated with decreased productivity in employees. The objective of this retrospective study is to assess the antimigraine medication use and associated resource utilization in employed patients. Patients with primary diagnosis of migraine or receiving antimigraine prescription drugs were identified from an employer-sponsored health insurance plan in 2010. Medical utilization and health care costs were determined for the year of 2010. Generalized linear regression was applied to evaluate the association between health care costs and the use of antimigraine medications by controlling covariates. Of 465 patients meeting the study criteria, nearly 30% that had migraine diagnosis were prescribed antimigraine medications, and 20% that had migraine diagnosis were not prescribed antimigraine medications. The remaining 50% were prescribed antimigraine medications but did not have migraine diagnosis. Patients with antimigraine medication prescriptions showed lower frequency of emergency department visits than those without antimigraine medication prescriptions. Regression models indicated an increase in migraine-related health care costs by 86% but decreases in all-cause medical costs and total health care costs by 42 and 26%, respectively, in the antimigraine medication use group after adjusting for covariates. Employed patients experienced inadequate pharmacotherapy for migraine treatment. After controlling for covariates, antimigraine prescription drug use was associated with lower total medical utilization and health care costs. Further studies should investigate patient self-reported care and needs to manage headache and develop effective intervention to improve patient quality of life and productivity.

## Introduction

Migraine is a chronic condition characterized by moderate to severe headaches. About 29.5 million Americans suffer from migraine, with an estimated 18% of women and 6% men directly affected [1–3]. The predominate (or primary) age to experience migraines is between 25 and 55, which coincides with an individual’s most active employment period [4, 5]. Consequently, migraine-attributable morbidity may adversely affect work productivity via absenteeism or presenteeism (compromised work productivity in the course of daily labor) [6]. Additionally, migraine sufferers may also experience compromised rest and leisure that indirectly affects labor productivity, and directly acerbates quality of life [7–9]. Studies suggest migraine-related morbidity, particularly, accounts for costs incurred via decreased productivity, with United States’ annual estimates between $13 billion and $17 billion [10–12]. Unfortunately, migraine is both under diagnosed and inadequately treated in the majority of patients. One study evaluated treatment patterns and health care use for chronic migraine in a general population, and demonstrated 87.6% of the patients with chronic migraine visited a health care provider to obtain appropriate care [13]. Nearly 32% of those patients received migraine-specific acute treatments and just 33% were using preventive medications. Another study considered migraineurs’ current patterns of health care use, and noted 48% of migraine suffers had seen a doctor for headache within the past year, 31% had never done so in their lifetimes, and 21% had not seen a doctor for headache for at least 1 year [1]. Of all the patients with migraine, 23% were treated with prescription drugs, and only 49% with over-the-counter medications only. Thus, previous studies suggest a care gap exists relative to evidence-based care strategies, and subsequent application in migraine management.

Although proper diagnosis is essential to optimal migraine management, increased burden may also be attributed to inadequate prescription drug therapy. Studies demonstrated improved medication use is associated with better clinical and economic outcomes in patients with chronic diseases. Specifically, unsustained statin use compromises benefits, such as reduced CHD risk, and creates substantial treatment costs attributed to otherwise preventable CHD events [14–17]. Balkrishnan’s study also found a strong association between decreased antidiabetic medication use and increased health care service utilization in elderly with type 2 diabetes [18]. Currently, many studies on migraine and associated outcomes rely upon data gleaned via patient self-report surveys [1, 13, 19–21]. Knowledge of migraine-associated health outcomes in patients enrolled in employer-sponsored health insurance programs is very limited. Insights gleaned from a population having a common baseline of assured care access may help clarify where and how suboptimal patient outcomes persist. It is unclear whether there is a relationship between migraine medication use and total medical utilization and costs, especially in employed patients. Clarifying this relationship may elucidate the impact of explicit chronicity on total medical costs, and subsequently clarify comprehensive care coordination opportunities. Health care administrative data is a readily available source of employees’ health care resource utilization, and may inform insights regarding clinical and economic outcomes. This study was designed to answer the following two primary questions: (1) What is the antimigraine prescription drug use pattern in patients enrolled in an employer-sponsored health plan? (2) How do the resource utilization and health care costs differ between patients receiving prescription drugs to treat migraine and those not receiving prescription drugs?

## Methods

### Data source and patient selection

De-identified claims data based on an employer-sponsored health plan were extracted by Advisory Board Company in Washington D.C. All patients with at least one primary diagnosis of migraine between January 1, 2010 and December 31, 2010 were identified by International Classification of Diseases, 9th revision (ICD-9) (346.xx). All patients receiving at least one prescription for a medication to treat migraine between January 1, 2010 and December 31, 2010 were also identified by therapeutic category in the database (Table 1). Patients were required to be enrolled in the employer-sponsored health plan continuously during the year of 2010. Patients enrolled as dependents were excluded from the study. Given the bidirectional influence of migraine and major depression described in previous studies [22, 23], we considered comorbid depression if patients presented at least one primary diagnosis of major depression or received at least one prescription for an antidepressant or antianxiety medication shown in Table 1 during the study period. All eligible patients were divided into two groups: patients who received antimigraine medications and those who did not.

**Table 1:** Medications for migraine and depression used by participants

Antimigraine medications	Antidepressants
Acetaminophen/caffeine/isometheptene mucate	Alprazolam
Acetaminophen/dichloralphenazone/isometheptene mucate	Alprazolam extended-release
Almotriptan malate	Amitriptyline hydrochloride
Dihydroergotamine mesylate	Amitriptyline-chlordiazepoxide
Eletriptan hydrobromide	Budeprion hydrochloride
Ergotamine-caffeine	Budeprion hydrochloride extended-release
Frovatriptan succinate	Budeprion hydrochloride sustained-release
Naratriptan hydrochloride	Citalopram hydrobromide
Rizatriptan benzoate	Clomipramine hydrochloride
Sumatriptan succinate	Clorazepate dipotassium
Sumatriptan succinate/naproxen sodium	Desipramine hydrochloride
Zolmitriptan	Desvenlafaxine succinate
	Diazepam
	Doxepin hydrochloride
	Duloxetine hydrochloride
	Escitalopram oxalate
	Fluoxetine hydrochloride
	Fluoxetine hydrochloride/olanzapine
	Fluoxetine pamoate
	Imipramine hydrochloride
	Lorazepam
	Mirtazapine
	Nortriptyline hydrochloride
	Oxazepam
	Paroxetine hydrochloride
	Paroxetine mesylate
	Phenelzine sulfate
	Trazodone hydrochloride
	Venlafaxine hydrochloride
	Venlafaxine hydrochloride extended-release

### Medical utilization

Total medical utilization with any conditions within 1 year was evaluated by hospitalization frequency, emergency department (ED) visits, and total numbers of outpatient and physician office visits. Patients with at least one time hospitalization or ED visit during the study period were categorized as hospitalization or ED visit during the study period. Medical utilization related to migraine was identified by primary diagnosis codes (346.xx).

### Health care costs

Health care costs were evaluated based on third party payer perspective. Reimbursement rates paid by the employer were used to compute annual total and migraine-related health care costs, including medical care and pharmacy costs. Medical care costs were defined as the sum of inpatient, ED, hospital outpatient, and physician office visit costs. Annual total health care costs were the sum of medical and pharmacy costs associated with any conditions during the 1-year study period. Migraine-related health care costs were identified by primary diagnosis code of migraine (346.xx) for medical care costs and by prescription drugs to treat migraine for pharmacy costs. Total migraine-related health care costs were the sum of migraine-related medical and pharmacy costs during the 1-year study period.

### Risk index (RI) and Care gap index (CGI) [24]

RI is an index value created by using D2Hawkeye risk modeling system to indicate the risk a member will become a catastrophic case, and is based on the number of comorbidities presented. Similarly, CGI is an index value indicating the degree to which a member complies with recommended care guidelines associated with their age and existing conditions. Both of these scores provided by the health insurance program are routinely used by a number of care management program directors and managers to identify specific patient groups that may particularly benefit from tailored care management programs. These two variables were included in our regression models as covariates for adjustment.

### Data analysis

Univariate analyses were used to compare patient characteristics, unadjusted medical utilization, and health care costs between the two groups, namely, those using antimigraine medications and those not using antimigraine medications. Chi-square tests were applied to analyze categorical variables and *t* tests were used for continuous variables. Nonparametric tests (Wilcoxon rank-sum test) were used to compare various health care costs between two groups.

Generalized linear regressions with log link and gamma distribution were considered to analyze the associations between health care costs and the use of antimigraine medications after adjusting for diagnosis of migraine, age, gender, comorbid depression, CGI, and RI. The level of significance was preset at *α* = 0.05. All statistical analyses were conducted by SAS 9.2 (SAS Institute, Cary, NC).

## Results

**Fig. 1:**
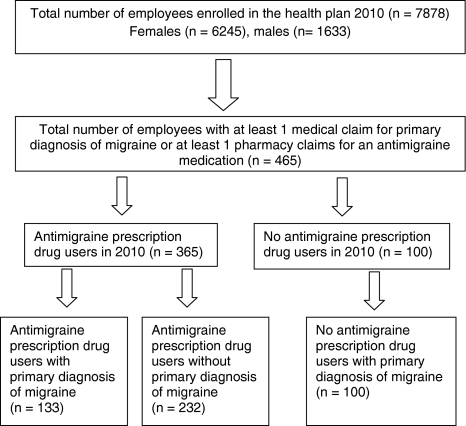
Patient selection from an employer-sponsored health insurance plan in 2010

Figure 1 and Table 2 display the patient selection and characteristics of our study population enrolled in an employer-sponsored health plan during the study period (January 1, 2011 to December 31, 2011). A total of 465 patients (5.9% of total employees enrolled in the employer-sponsored health insurance plan in 2010) met the study inclusion and exclusion criteria, with an average age of 43.5 years. Females accounted for nearly 95% of the study population; two-thirds of the patients had depression diagnosis or used antidepressants during the study period. Of the patients receiving antimigraine medications, 64% did not have migraine diagnosis. In contrast, 43% of patients diagnosed with migraines, did not take antimigraine medications.

**Table 2:** Characteristics of the study population (*n* = 465)

Variable	All patients (*n* = 465)	Antimigraine drug users (*n* = 365)	No antimigraine drug users (*n* = 100)	*p* value
Age (years), mean (SD)	43.5 (10.9)	43.8 (10.9)	42.3 (11.0)	0.210
Gender female, *n* (%)	433 (93.1)	338 (92.6)	95 (95.0)	0.402
CGI, mean (SD)	2.4 (2.3)	2.4 (2.3)	2.7 (2.2)	0.150
RI, mean (SD)	17.6 (10.5)	17.7 (10.3)	17.5 (11.3)	0.861
Depression, *n* (%)	307 (66.0)	240 (65.8)	67 (67.0)	0.816
Diagnosis of migraine, *n* (%)	233 (50.1)	133 (36.4)	100 (100)^a^	<0.001

*CGI* care gap index, *RI* risk index, *SD* standard deviation

^a^Patients who neither used antimigraine drugs nor had migraine diagnosis were not included in the study

Table 3 compared unadjusted medical utilization and health care costs between patients using and not using antimigraine medications. Patients receiving antimigraine medications evidenced 12.3 and 7.2% fewer all-cause and migraine-related ED visits, respectively, as compared to those not receiving antimigraine medications. Groups using antimigraine medication also evidenced fewer outpatient and office visits. Pharmacy cost for antimigraine medication use accounted for 78% of total migraine-related costs. An increase in all-cause pharmacy cost by $1,010 and decrease in all-cause medical cost by $3,407 were shown in the antimigraine medication use group.

**Table 3:** Medical utilization and health care costs of the study population by antimigraine medication use (*n* = 465)

Variable	Antimigraine drug users (*n* = 365)	No antimigraine drug users (*n* = 100)	*p* value
All-cause medical utilization			
Hospitalization, *n* (%)	39 (10.7)	15 (15.0)	0.233
ED visits, *n* (%)	83 (22.7)	35 (35.0)	0.013
Total number of outpatient visits, mean (SD)	13.2 (11.9)	15.3 (15.6)	0.220
Migraine-related medical utilization			
Hospitalization, *n* (%)	0	3 (3.0)	<0.001
ED visits, *n* (%)	14 (3.8)	11 (11.0)	0.005
Total number of outpatient visits, mean (SD)	0.8 (1.8)	1.4 (1.2)	<0.001
All-cause health care costs ($), mean (median)			
Medical	7,043.7 (1921.4)	10,450.6 (3352.0)	0.019
Pharmacy	2,827.5 (1408.1)	1,817.3 (753.1)	<0.001
Total	9,871.2 (4648.1)	12,267.9 (5267.3)	0.633
Migraine-related costs ($), mean (median)			
Medical	149.3 (0)	634.1 (100.3)	<0.001
Pharmacy	516.1 (201.9)	0	<0.001
Total	665.4 (283.5)	634.1 (100.3)	0.001

*ED* emergency department, *SD* standard deviation

Table 4 presents associations between various health care costs and the use of migraine medications after adjusting for diagnosis of migraine, age, gender, CGI, RI, and comorbid depression. After translating the log-transformed coefficients based on generalized linear regressions, we found even though patients receiving antimigraine medications showed increased migraine-related health care costs by 86%, all-cause medical costs and total health care costs were reduced significantly by 42 and 26%, respectively, when all the other covariates remained constant.

**Table 4:** Associations between health care costs and the use of antimigraine medications

Independent variables	Estimated coefficient (SE)		
	Dependent variable: log (total all-cause health care costs)	Dependent variable: log (all-cause medical costs)	Dependent variable: log (total migraine-related costs)
Antimigraine medication use (yes vs. no)	−0.30 (0.12)**	−0.55 (0.17)***	0.62 (0.18)***
Diagnosis of migraine (yes vs. no)	−0.30 (0.10)**	−0.37 (0.14)**	1.17 (0.15)***
Age (years)	−0.01 (0.28)*	−0.02 (0.01)**	0.02 (0.01)**
Gender (female vs. male)	0.21 (0.18)	0.17 (0.23)	−0.15 (0.25)
CGI	0.05 (0.02)*	0.05 (0.03)	0.05 (0.03)
RI	0.08 (0.01)***	0.08 (0.01)***	0.01 (0.01)
Comorbid depression (yes vs. no)	0.15 (0.10)	0.19 (0.13)	0.04 (0.14)

*CGI* care gap index, *RI* risk index, *SE* standard error

* p < 0.05, ** *p* < 0.01, *** *p* < 0.001

## Discussion

The purpose of this study was to evaluate the use pattern of migraine prescription drugs and its effect on the health care costs in patients enrolled in an employer-based health care program. Our study results indicated that nearly 45% of the patients with diagnosis of migraine did not receive appropriate pharmacotherapy and that nearly 65% of the patients who received antimigraine medications did not have migraine diagnosis. This study also found that migraine medication costs were the major proportion of migraine-related costs in patients, but total cost saving was shown in the patients due to significantly decreased medical costs. These findings may underscore the importance of improving current evidence-supported strategies (such as appropriate pharmacotherapy and prophylactic regimens) and incorporating adjunct interventions, such as patient self-management. These efforts may improve the quality of life and productivity in employed patients with migraine.

Pharmacotherapy, especially the triptan drug class, accounts for the most direct costs relative to migraine care; given the expense, it is critical to appropriately administer and facilitate optimal use. Clouse’s study compared health care use and associated costs in patients with migraine and patients without migraine in a managed-care organization. Clouse’s study [25] indicates patients with migraine had greater morbidity in general, and incurred 64% greater costs in health care resource use as compared with patients without migraine. Thus, migraine medication prescription and use may mitigate total health care costs, and have favorable implications for the third party payers. This finding may underscore the significance of our findings, as increased pharmacy cost resulting from antimigraine medications may by justified by total medical care cost reduction in patients with migraine (after adjusting for covariates).

Patients report headache as one of the most commonly reported reasons for ED visits [26, 27]. These visits may represent an annual cost ranging from $600 million to nearly $2 billion [12]. Our study also revealed that the percentage of patients with at least one migraine-related or all-cause ED visit was 7.4 or 12.3% lower in the patients using antimigraine medications than those not using antimigraine medications. Thus, lower medical utilization and costs could be expected in patients subsequent to medication initiation. In addition, using agents for migraine prophylaxis might be considered to manage the disease and control medical costs. Migraine prophylaxis is aimed at preventing frequent attacks, including migraine severity and length that often incurs high costs for pain relief, diagnostic services, and medical care. Wertz et al. [28] reported that headache-related resource utilization and costs were significantly lowered after initiation of preventive migraine treatment in a sample of a large managed-care population. Therefore, appropriate preventive therapy may reduce the disease burden and medical utilization.

Our study also suggests suboptimal migraine therapy, which is consistent with previous report. Among patients with migraine diagnosis, only 57% took antimigraine medications. Notably, although pharmacy cost was reduced without taking prescription drugs, total health care costs were increased due to heavy costs on medical utilization. Table 3 showed that those not using antimigraine drugs presented higher frequency of ED visits and total number of outpatient visits related to migraine. These results suggested that poor health outcomes and disease management might be associated with inadequate antimigraine medication use. Based on this evidence, there is a need to improve the quality of migraine care, and particularly provide physicians and patients with therapeutic options that are effective and well tolerated. Moreover, we also found that two-thirds of all patients in the study has depression diagnosis or used antidepressant medications. Studies showed that depression and migraine are highly correlated and might be bidirectional. That is, migraine may cause psychiatric conditions, vice versa or both [23, 29]. Both of the diseases significantly decrease health-related quality of life [8]. Thus, assessing depressive disorders in migraine patients may augment migraine care quality.

These findings, derived from employer-sponsored claims data, suggested antimigraine medication use may be associated with reduced total medical utilization and health care costs. The study results indicate opportunities to understand self-reported care and needs of employees with headache. We may further investigate migraineurs’ quality of lives and productivity via survey tools. Informed by the results of this study, we may develop intervention strategies to mitigate both direct (health care plan costs) and indirect (loss productivity and absenteeism) cost. More importantly, our results indicate a manner to improve migraine care, thereby increasing employees’ the quality of care via treatment optimization and targeted implementation.

Although this study provides evidence regarding the associations among antimigraine medication use, diagnosis of migraine, and resource utilization, we concede some study limitations. First, the retrospective cross-sectional study design restricts the ability to establish causal inferences related to antimigraine medication use. Second, some of these patients could use over-the-counter (OTC) medications instead of prescription drugs to manage the headache, subsequently biasing our claims-based analyses. Additionally, some patients receiving antimigraine medications without migraine diagnosis code could occur in the administrative data. Third, we used a diagnosis of depression or antidepressant medication use as a comorbid depression. It is possible that some of these patients received antidepressant agents for the treatment of conditions other than depression. Finally, the claims data provide information on prescribing patterns only rather than on actual medication use. Therefore, we suggest further investigations include patient self-reported medication use using a survey tool.

## Conclusions

Noted limitations notwithstanding, the study results suggest inadequate pharmacotherapy in migraine treatment. After controlling for covariates, antimigraine prescription drug use was associated with lower total medical utilization and health care costs. This suggests appropriately managing a specific chronicity, potentially inflating disease-specific expenditures, can favorably impact total health care costs. Further studies should investigate patient self-reported care and needs to manage headache and develop effective intervention to improve patient quality of life and productivity.
